# Determining the Age of Terrace Formation Using Luminescence Dating—A Case of the Yellow River Terraces in the Baode Area, China

**DOI:** 10.3390/mps3010017

**Published:** 2020-02-20

**Authors:** Jia-Fu Zhang, Wei-Li Qiu, Gang Hu, Li-Ping Zhou

**Affiliations:** 1 MOE Laboratory for Earth Surface Processes, College of Urban and Environmental Sciences, Peking University, Beijing 100871, China; hugang@ies.ac.cn (G.H.); lpzhou@pku.edu.cn (L.-P.Z.); 2 Faculty of Geographical Science, Beijing Normal University, Beijing 100875, China; qiuweili@bnu.edu.cn; 3 Institute of Geology, China Earthquake Administration, Beijing 100029, China

**Keywords:** terrace strath and tread ages, luminescence dating, deposition rate, age-depth model, Yellow River terrace

## Abstract

Dating fluvial terraces has long been a challenge for geologists and geomorphologists, because terrace straths and treads are not usually directly dated. In this study, the formation ages of the Yellow River terraces in the Baode area in China were determined by dating fluvial deposits overlying bedrock straths using optically stimulated luminescence (OSL) dating techniques. Seven terraces (from the lowest terrace T1 to the highest terrace T7) in the study area were recognized, and they are characterized by thick fluvial terrace deposits overlaid by loess sediments. Twenty-five samples from nine terrace sections were dated to about 2–200 ka. The OSL ages (120–190 ka) of the fluvial samples from higher terraces (T3–T6) seem to be reliable based on their luminescence properties and stratigraphic consistency, but the geomorphologic and stratigraphic evidence show that these ages should be underestimated, because they are generally similar to those of the samples from the lower terrace (T2). The formation ages of the terrace straths and treads for the T1 terrace were deduced to be about 44 ka and 36 ka, respectively, based on the deposition rates of the fluvial terrace deposits, and the T2 terrace has the same strath and tread formation age of about 135 ka. The incision rate was calculated to be about 0.35 mm/ka for the past 135 ka, and the uplift rate pattern suggests that the Ordos Plateau behaves as a rigid block. Based on our previous investigations on the Yellow River terraces and the results in this study, we consider that the formation ages of terrace straths and treads calculated using deposition rates of terrace fluvial sediments can overcome problems associated with age underestimation or overestimation of strath or fill terraces based on the single age of one fluvial terrace sample. The implication is that, for accurate dating of terrace formation, terrace sections should be systematically sampled and dated.

## 1. Introduction

Fluvial terrace deposits and landforms can provide important information about river incision, tectonic activity, climate change and archaeological traces of hominid activity [1,2,3,4,5], and terraces record different stages of fluvial evolution and sedimentation. Yet, understanding the formation of terraces is a perennial problem in geomorphology and has been hampered by the exact timing of terrace formation. Determining the formation age of terraces is a challenge for geomorphologists and geologists. Recently, the successful application of optically stimulated luminescence (OSL) dating techniques [6,7,8] to fluvial sediments [9,10] has made it possible to establish the chronology of terraces by dating fluvial sediments atop terrace straths [11,12,13,14,15]. Conventionally, only a single age is assigned to a terrace, but this may not reflect actual fluvial processes [16,17]. The development of OSL techniques allows dating a series of samples from terrace deposits by systematic sampling, which helps to establish the chronology of deposition and then deduce the formation ages of terrace straths and treads [18,19,20].

The investigations of Yellow River fluvial terraces have become one of the most important topics in Chinese geomorphology and are also of interest to researchers in sedimentology, engineering, hydrology and archaeology. The fluvial terraces in the Jinshaan Canyon in the middle reaches of the Yellow River have been widely investigated and dated mainly using luminescence and paleomagnetic dating methods [18,19,21,22,23,24,25,26]. However, the dates of the terraces obtained are not enough to construct the precise chronology of a series of terraces in the region. This is further complicated by the fact that some terraces with the same elevations above the modern river have different formation ages, because their formations are controlled by knickpoint migration [19,25]. Therefore, it is necessary to determine the ages of more Yellow River terraces in different localities of the region. On the other hand, one sample for OSL dating is often collected from terrace deposits in fluvial terrace investigations [21], and its age is usually assumed to be the terrace formation age. This assumption may be incorrect, and its validity needs to be tested. In this study, we first identified terraces in the two banks of the Yellow River in the Baode area in the middle reaches of the river, and terrace deposits were systematically sampled for OSL dating, i.e., a series of samples were collected from channel facies and floodplain facies and overlying loess deposits on terraces. The reliability of their ages was evaluated based on the internal consistency of the OSL ages and the lithostratigraphic and geomorphological consistency in the OSL ages, and the formation ages of terrace straths and treads were then inferred.

## 2. Geological and Geomorphologic Setting

**Figure 1:**
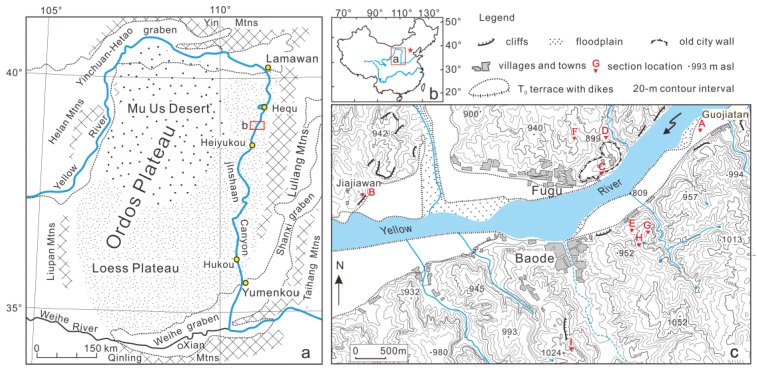
(**a**,**b**) Map showing the Yellow River course along the margins of the Ordos Plateau and location of the study area (Baode); (**c**) topographic map showing the localities of the sections (A–I) shown in Figure 2. The topographic map with contour intervals of 20 m was constructed based on a Chinese 1:50,000 topographic map (unpublished).

The Ordos Plateau, with an area of 320,000 km^2^ located in the western part of the North China block, is a relatively stable rigid block (Figure 1a) [27,28]. It is bounded by a series of marginal faults and orogenic belts [29,30,31,32,33,34]. The basement of the plateau is composed of Archean and Proterozoic metamorphic rocks that are overlain by Cambrian and Ordovician marine carbonates with the lack of Silurian and Devonian strata. Carboniferous marine limestone and fluvial-deltaic sandstone are overlain by fluvial Permian strata. Triassic and Jurassic strata are composed of fluvial and lacustrine deposits, which are overlain by Cretaceous fluvial and eolian redbeds. The Ordos block evolved from a basin to a plateau during the late Miocene–Pliocene, which influences the environmental effect on the topography of the area [35]. Geomorphologically, the Ordos Plateau is surrounded by the Yin Mountains to the north, Luliang Mountains to the east, Qinling Mountains to the south, Liupan and Helan Mountains to the west (Figure 1). The arc-shaped Yinchuan–Hetao graben system along the northwestern and northern margins of the block and the S-shaped Weihe-Shanxi graben system along the southeastern margins bound the plateau [29,36]. The plateau is covered by Quaternary eolian sediments. The northern part, the Mu Us Desert, is mantled by dune sands, and the southern part, the Chinese Loess Plateau, is covered by loess/paleosol deposits.

The Yellow River (called Huanghe in Chinese), well-known for its tremendous sediment load, originates in the northeast of the Tibetan Plateau. In its middle reaches, the river flows along the northeastern and northern margins of the Ordos Plateau and cuts through the eastern plateau at an average elevation ranging from 1000 to 1500 m above sea level (asl), from Lamawan in the north to Yumenkou in the south (Figure 1a). The river then turns east and finally overflows into the Bohai Sea. The river downcutting between Lamawan and Yumenkou leads to the formation of a deep and narrow gorge called Jinshaan Canyon, which connects the Yinchuan-Hetao graben on the north and the Weihe graben on the south. A series of strath terraces along the banks of the canyon have been found based on the presence of fluvial deposits and bedrock strath. The terrace treads are covered by loess with various thicknesses.

## 3. Methodology

### 3.1. Field Work

As shown in Figure 1a, the two banks of the Yellow River in the study area are covered by loess/paleosol deposits. This implies that the original stair-stepped topography of the fluvial terraces in the area is not directly observed, and the situation is further complicated by the modification of the loess landscape due to soil erosion. The fluvial terraces cannot be mapped from topographic maps, satellite images or air photos or even identified in the field based on surface landform. In this case, the identification of remnants of the terraces was only conducted by observing fluvial sediments exposed in natural outcrops or road-cuts in the field. This means that the terraces were found as isolated remnants. The heights of terrace strath (bedrock surface) and tread (here the “tread” is referred to as the near-horizontal top surface of fluvial deposits, including channel or overbank facies resting on terrace straths) above the modern river level and the thickness of the terrace deposits were measured using a total station and a tape measure. The precise locations and altitude of the exposures were recorded using a hand-held global positioning system (GPS) receiver with a barometric altimeter (Garmin GPSmap 60CSx) with an accuracy of ±~3 m in altitude. Stratigraphy and lithology of the exposures were described in detail.

**Figure 2:**
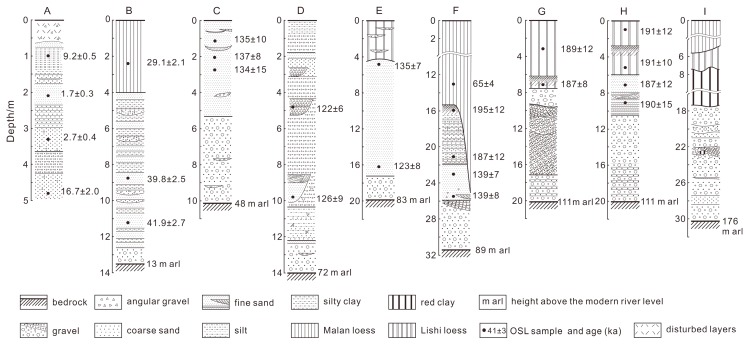
Stratigraphic columns of the terrace deposits in outcrops (A–I) marked in Figure 1c, and the positions of optically stimulated luminescence (OSL) samples and ages in ka. The elevations of bedrock surfaces (strath surface) above the modern river are displayed.

A systematic sampling strategy was adopted for dating the sediments on terrace straths using OSL techniques. Systematic sampling means that a series of samples are taken from fluvial and loess deposits on a terrace, and their OSL ages are used to construct an age-depth model for the terrace deposits. The model helps us to evaluate the reliability of OSL ages obtained for the terraces and to explain the history of river incision. The formation ages of the strath and tread for a terrace can be inferred from the age-depth relationship. OSL samples were taken from each unit of terrace deposits and overlying loess/paleosol (see Figure 2). The samples were collected by hammering 3.5-cm-diameter and 30-cm-long stainless-steel tubes horizontally into freshly cleaned exposures. The tubes were wrapped with aluminum foil and adhesive tape in order to prevent further exposure to light and moisture loss.

### 3.2. Optical Dating

#### 3.2.1. Equivalent Dose Measurements

The samples for OSL measurements were prepared in our dark room with a dim red light at the Peking University, Beijing, China. Coarse- and fine-grained quartz were extracted from sand- and silt-sized samples using the procedures in our laboratory, respectively [37,38]. In the laboratory, the light-exposed ends (about 2–3 cm of sediment) of a sample tube were first removed, and this removed material was used for dose-rate analysis. The remaining material from the middle of the tube was treated with 10% hydrochloric acid to dissolve carbonates and then 30% hydrogen peroxide to remove organic material, respectively. For sand-sized samples, the material was then washed with water to eliminate finer grains, followed by drying and sieving to select coarse grains (grain sizes for each sample are listed in Table 1) for luminescence measurements. The coarse-grained quartz was obtained by immersing the sieved sample in 40% HF for 40 or 80 min and then 10% HCl to remove feldspar contaminants. For fine-grained samples, the samples were then deflocculated using a dilute sodium oxalate solution, and polymineral fine grains (4–11 μm) were isolated by settling the polymineral fine silt fraction in the solution. Fine-grained quartz was obtained by treating the polymineral extracts with silica-saturated fluorosilicic acid (H_2_SiF_6_) at room temperature to dissolve feldspars, amorphous silica and other contaminant minerals, followed by a treatment with 10% HCl to remove any fluorides produced. The purity of the quartz extracts was checked by infrared stimulation. The results showed that the infrared-stimulated luminescence signals were negligible, indicating feldspar contaminants were almost entirely removed. The chemically purified quartz was prepared for luminescence measurements by settling the fine grains in acetone onto 0.97-cm-diameter aluminum discs or mounting the coarse grains as a monolayer on 0.97-cm-diameter aluminum discs with the grains covering the area with a diameter of ~5 mm (medium aliquots) using silicone oil as an adhesive.

The improved single-aliquot regenerative dose procedure (SAR) [39,40] was used to measure the single-aliquot equivalent dose (De) of the quartz extracts at Peking University. The regenerative beta doses used in the SAR procedure included a zero dose used for monitoring recuperation effects and a repeat of the first regeneration dose used to check the reproducibility of the sensitivity correction (i.e., recycling ratio). Based on the results of the preheat plateau and dose recovery tests (see below), the preheat temperature was set to 220 °C or 260 °C, and the cut-heat temperature to 160 °C. OSL signals were measured for 40 s at 125 °C. In addition, a 20-s IR stimulation at room temperature before each OSL measurement was carried out to remove the possible effect of feldspar contamination [38], although IRSL signals were negligible, and a 40-s blue light stimulation at 280 °C at the end of each cycle was also carried out for reducing recuperation. The signals were analyzed using late background subtraction (the intensity of the initial 0.16 s minus a background (normalized to 0.16 s) from the last 3.2 s), and the value of De was estimated by interpolating the sensitivity-corrected natural OSL onto the dose-response curve using the Analyst software [41]. The error on individual De values was calculated using the counting statistics and an instrumental uncertainty of 1.0%.

All luminescence measurements, beta irradiation and preheat treatments were carried out in automated Risø TL/OSL (DA-15 and DA-20) readers equipped with a ^90^Sr/^90^Y beta source (the Risoe National Laboratory, Denmark Technical University, Denmark) [42]. Blue light LED (470 ± 30 nm) stimulation was used for quartz OSL measurements and an IR laser diode (830 ± 10 nm) stimulation for scanning feldspar contamination and for feldspar IRSL measurements. Luminescence was detected by an EMI 9235QA photomultiplier tube with two Hoya U-340 filters (290–370 nm) in front of it.

#### 3.2.2. Dose Rate Determination

The uranium and thorium contents of samples L782–787 were determined using thick-source alpha counting (a Littlemore low-level alpha counter 7286 with 42-mm-diameter ZnS screens) (Littlemore Scientific, UK), and other samples were analyzed using the neutron-activation-analysis (NAA) for U, Th and K contents. The K content of all samples was also measured using flame photometry. The present-day water contents (ratio of mass of water/dry-sample [6]) of all the samples were measured in the laboratory to be 1.0%–8.4%, with an average of 3.1±0.5%, by weight. These samples were taken from the subsurface position of the sections, and they have been partly dried due to exposure to air before sampling. These values are clearly not to be representative of the long-term water contents in the natural conditions during most of the burial history. In this case, the water contents used for dose rate calculation were assumed and taken as 5% for sand, 10% for loess and silt sediments (overbank deposits) and 15% for paleosol. A relative uncertainty is taken as 20% to the long-term water content values. This large uncertainty on the water content should cover the water content fluctuations during burial. An alpha efficiency factor (*a*-value) of 0.038 ± 0.003 for quartz [43] was used to calculate the alpha contribution to the total dose rate. Based on the above measurements, the effective dose rates and ages were calculated using the online dose rate and age calculator DRAC v1.2 [44], in which cosmic ray contribution and conversion factors [45] are involved, and the alpha [46] and beta [47] grain attenuation factors were used.

## 4. Results

### 4.1. Terraces and Deposits

**Figure 3:**
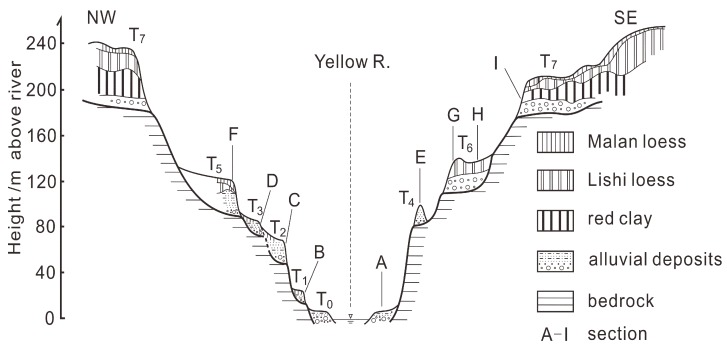
Schematic composite cross-section across the Jinshaan Canyon at the Baode area showing the Yellow River terrace sequence and fluvial and overlying loess/paleosol deposits, which are shown in details in Figure 2 (here, the letters refer to the number of sections in Figure 2).

A total of nine exposures of fluvial sediments in the study area were found and observed. Their locations (numbered A to I) are marked in Figure 1c, and their detailed lithology are shown in Figure 2. It can be seen that the sections (exposures) are mainly composed of fluvial sediments called terrace deposits and overlying eolian sediments. The fluvial sediments consist of channel gravels and sands capping the strath surfaces and overbank silt or silty clay overlying the channel deposits. The top eolian layers composed of loess, paleosol or red clay were deposited after paleo-floodplains were completely abandoned. The bedrock surfaces (strath) of Sections B–I were presented, and their elevations range from about 13 m to 176 m above the modern river level (arl) (Figure 2). Based on the elevation of the straths, Exposures B–I are considered to represent seven terraces, from the lowest T1 terrace with the elevation of about 13 m arl and the highest T7 terrace with the elevation of 176 m arl. Accordingly, a schematic composite across-section of the terraces was generated based on the elevations of the terraces and their spatial distribution and is shown in Figure 3. Note that T0 (Section A) is referred to the high floodplain of the river. Exposures (Sections) G and H have the same elevation of strath (111 m arl) and belong to the T6 terrace. It is noted that the effect of the variations in river gradients on terrace height in the study area is negligible. 

The high floodplain (T0) is composed of sand interbedded with gravel and laminated silty (Figure 2A), and the thickness of the fluvial deposits is more than 4.5 m. The top layer of this section was disturbed by human activities. The thickness of the terrace deposits, including channel facies (gravels and sands) and overbank facies (silts), for the eight sections ranges from ~10 to ~16 m. The fluvial gravels have moderate-to-high sphericity and are well-rounded. The gravel layers have a thickness ranging from 1 to 12 m, within which, crude imbrication is present locally, such as in Section G. The overbank silts overlying gravel layers are characterized by thin horizontal bedding. Some silty layers are interbedded with sand or gravel or sand lenses. The thickness of the silt layers varies from 0 to 8 m. The top loess/paleosol deposits are characterized by massive structure and vertical joints. On the highest terrace (T7, Section I), the red clay deposits were found between the top loess and fluvial sandy gravels resting on the bedrock. It is noted that the pre-Quaternary red clay deposits [48,49] are far beyond the upper limit of luminescence dating. Therefore, the T7 terrace is not sampled for OSL dating. The bedrock mainly comprises Triassic sandstone with a horizontal bedding, and the surface of bedrock is loose because of weathering. As shown in Figure 2, a total of 25 samples for OSL dating were taken from sections A to H. The information about the samples are listed in Table 1, and their positions are also shown in Figure 2. 

### 4.2. OSL Ages

**Figure 4:**
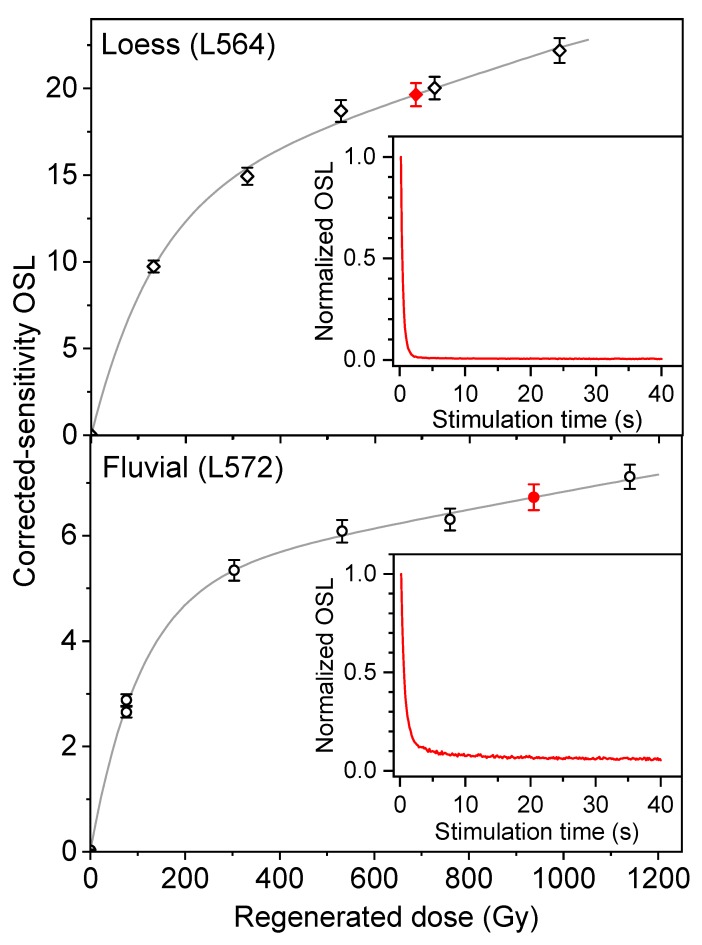
Dose response curves for fine quartz grains from loess sample (L564) and coarse quartz grains from fluvial sand sample (L572). The two curves are fitted by the functions of y = 13.8(1−exp(−(x + 0.139)/136) + 0.00869x and y = 3.17(1−exp(−x/83.3)) + 4.4(1−exp(−x/406)) + 0.0406, respectively. The filled diamonds and circles represent the corrected sensitivity natural signals. The insets show the decay curves for the natural signals from the two quartz samples. Note that the signals were normalized to unity at the first point.

Two dose-response curves (DRC) and two typical OSL decay curves for a fine quartz aliquot from a loess sample from the T6 terrace and a coarse quartz aliquot from a fluvial sample from the T5 terrace are shown in Figure 4. The decay curves demonstrate that the quartz OSL signals are easily bleached and dominated by fast components. The dose response curves are well-fitted with a double-saturating exponential function or a saturating exponential plus linear function. The recycling ratios are close to unity, and the recuperation values are less than 0.5%. The DRCs for all the samples are very similar, and the natural signals are apparently not close to saturation.

**Figure 5:**
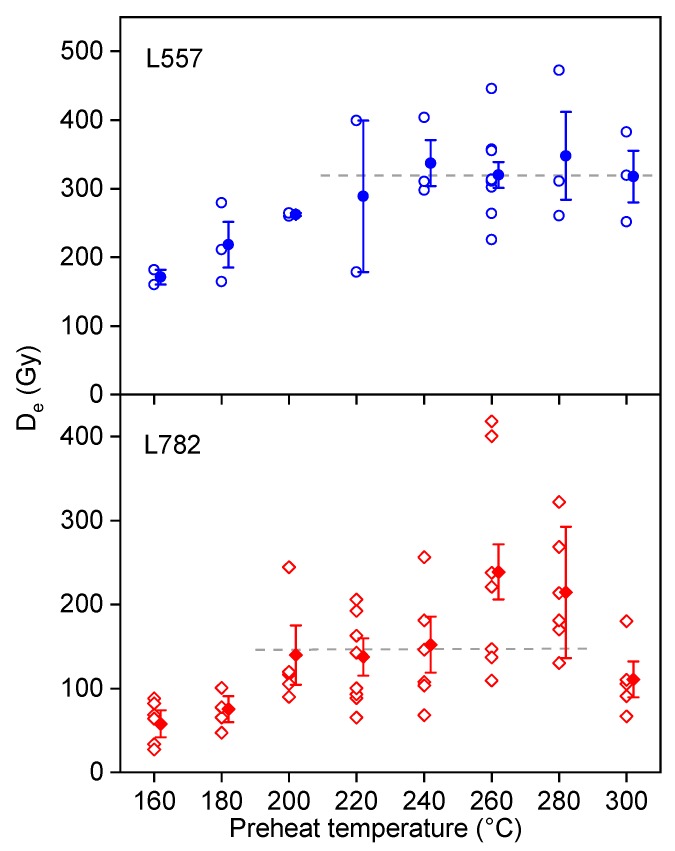
Dependence of De on preheat temperatures for samples L557 and L782. The open circles and diamonds represent the values of individual aliquots, and the filled circles and diamonds represent the average with their associated errors (one standard error).

**Figure 6:**
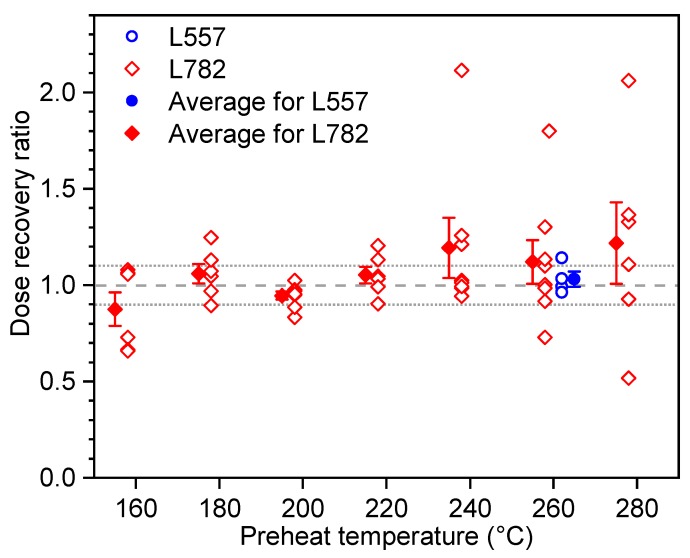
Plot of dose recovery ratios as a function of preheat temperatures for sample L557 and L782. The dose recovery ratios were obtained by dividing the recovery De values by the given doses (see text for details). The open circles and diamonds represent the values of individual aliquots, and the filled circles and diamonds represent the average with their associated errors (one standard error).

Preheat plateau and dose recovery tests were carried out on two samples to find the most suitable preheat temperature in the SAR procedure for our samples. Preheat plateau tests were performed using the SAR procedure with different preheat temperatures ranging from 160 °C to 300 °C at an interval of 20 °C. The results shown in Figure 5 demonstrate that the De values are independent of preheat temperatures, at least between 220 °C and 280 °C, for both samples. Dose recovery tests were performed on the same samples to further confirm the results of the preheat plateau tests. After removing the natural OSL signals by exposing aliquots to blue light within the readers at room temperature for 40 s, the residual OSL signals were examined by a second 40-s OSL measurement ~10,000 s after bleaching, and no detectable OSL signals could be observed. The aliquots were then irradiated with a laboratory beta dose approximately equal to the natural dose (De) of the sample. This artificial dose (given dose) was then taken as unknown, and the aliquots were treated as “natural samples”. After a storage of at least 10 h, the irradiated aliquots were then measured using the SAR procedure with the preheat temperatures of 160–280 °C with an interval of 20 °C for a relatively young sample (L782) from the T1 terrace and with the temperature of 260 °C for a relatively old sample (L557) from the T4 terrace. The dose recovery ratios (ratio of measured dose to given dose) were plotted as a function of the preheat temperature and shown in Figure 6. It can be seen that the average dose recovery ratios for each temperature are close to unity between the preheat temperatures of 180 °C and 280 °C for sample L782, and at 260 °C for sample L557, respectively. Based on the above results, the preheat of 220 °C for 10 s was adopted for the samples from the T1 terraces and 260 °C for 10 s for the samples from the higher terraces.

The dating results are summarized in Table 1, in which the arithmetic means and weighted means of individual De estimates are presented. The latter values and the overdispersion (OD) values are obtained using the “central age model” (CAM) of Galbraith et al. [50]. For most of the samples, their unweighted average De values are larger than their CAM values. It can be seen that the OD values for the sand samples from section A for the T0 floodplain vary from 53% to 72%, which are much larger than those (13%–31% with an average of 22.9% ± 1.5%) for the terrace samples.

**Figure 7:**
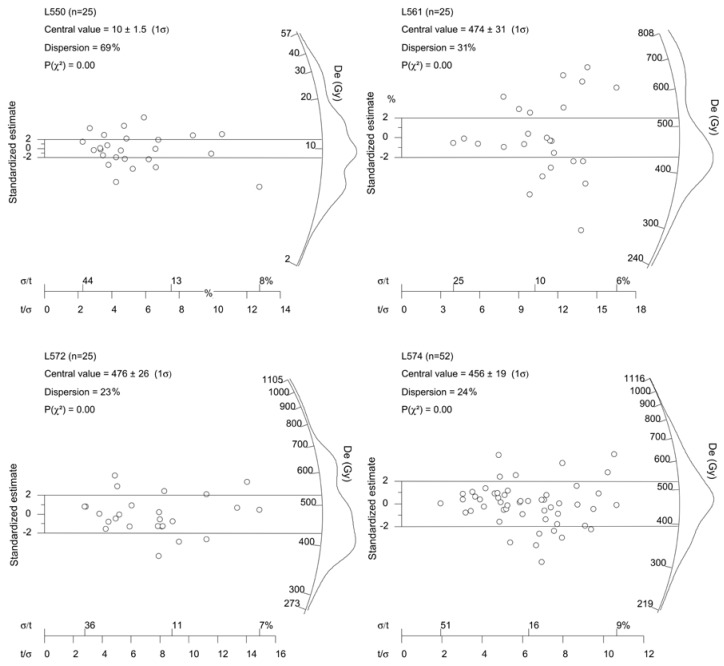
Abanico plots [51] showing the De distribution of four representative samples. The plots were generated using the RadialPlotter software (version 9.5) [52]. An abanico plot is the combination of both radial and kernel density estimate (KDE) plots [53]. It shows a visual correlation between De errors (radial plot) and De frequency distribution (KDE, on the z-axis of the radial plot). σ/t and t/σ refer to relative error (%) and precision, respectively.

Representative De distributions shown as abanico plots [51] are presented in Figure 7. Here, OSL ages were obtained by dividing the CAM De values by dose rates, and the age values are also shown in Figure 2

## 5. Discussion

### 5.1. Reliability of OSL Ages

As mentioned above, the good luminescence characteristics of the studied samples for the SAR protocol indicate that the SAR protocol is suitable for our samples [40]. However, the bleaching of fluvial sediments prior to burial for young samples may be problematic and which are generally evaluated by the OD values of their single-grain or single-aliquot De distributions. The three sand samples (L549, 550 and 551) from section A have large OD values (53%, 69% and 72%, respectively), indicating a large De scatter. The comparison with the global average value of 9 ± 3% published for well-bleached large-sized aliquots [54] implies that these three samples were poorly bleached at the time of deposition. Their CAM De values are 47.4 ± 5.6, 10.0 ± 1.5 and 5.0 ± 0.8 Gy, respectively; corresponding to the OSL ages of 16.7 ± 2.0, 2.7 ± 0.4 and 1.7 ± 0.3 ka, they are in stratigraphical order. Even if we assume that the De values of 10.0 ± 15 and 5.0 ± 0.8 Gy are the residual doses of the two samples at the time of deposition, the corresponding ages of 2.7 ± 0.4 and 1.7 ± 0.3 are similar to the age errors (one sigma) of the two sand samples (L551 and 550) from the T1 terraces. Their OSL ages are 39.8 ± 2.5 and 41.9 ± 2.7 ka, respectively (Table 1). Actually, previous investigations have demonstrated that the residual dose of modern fluvial sand samples from the middle reaches of the Yellow River are only 0.1 to 2.4 Gy, and have a large scatter, with OD values up to 90% [18,20,55]. The small residual doses of the modern fluvial sand samples are also confirmed by those of modern analogues from different rivers in the world (e.g., [56,57,58,59]). These suggest that the effect of the residual dose on the old samples from the terrace deposits in this study are insignificant. It is noted that the silty clay sample (L552) from the top of section A was much overestimated based on the comparison with the OSL ages of the underlying sand samples. That some fine grains from the Yellow River were relatively poorly bleached at deposition time is supported by the residual dose of the modern samples [20,55]. We deduced that fine grains were derived from nearby loess deposits due to storm and gravitational erosion [60]. Some grains were transported as aggregates, and there is not enough time to expose to sunlight because of near-distance transport from the source areas and rapid deposition. Relative to the large OD values of the samples from the floodplain of section A, the terrace deposits have smaller OD values of 12–31%. For example, there are 52 aliquots of sample L573 measured, and the sample exhibits log-normal De distribution with the OD value of 24% (Figure 7). The relatively large OD values may be attributed to the variations in intrinsic brightness among the individual aliquots and the large De values of the samples [15,61,62,63,64] and beta microdosimetry [65]. The difference in luminescence properties between coarse grains may be attributed to the different sources of the sediments associated with the Yellow River [55,60,66,67], including some grains from the local weathered sandstone bedrock [68]. In summary, the effect of the residual dose on the terrace samples in this study can be neglected.

As shown in Table 1, the samples from the T2–T6 terraces have the De values (arithmetic mean) ranging from 228 to 643 Gy, and most of them are >320 Gy. The reliability of the De values obtained in the high-dose region of dose-response curves has been debated [69,70,71,72,73,74]. For practical purposes, the 2D0 value (characteristic saturation dose) of a dose response curve fitted with a single saturating exponential function is usually used as a criterion to evaluate the upper limit for precise age determination [40,74]. However, the D0 value has been found to be varied with the size of the maximum regeneration dose [74]. This is also the case for D01 and D02, when a double-saturating exponential function is applied [75]. In this study, the two typical dose response curves fitted with a double-saturating exponential function and a saturating exponential plus linear function, respectively, and shown in Figure 4 demonstrate that the two curves are not fully saturated at the dose of up to 1000 Gy, which are larger than the De values obtained for our samples. This means that the reliability of the OSL ages for our samples cannot be evaluated only on the basis of the shapes of the dose-response curves. Furthermore, there are no independent age controls in this study. In this case, the reliability of the OSL ages obtained for the terrace samples are assessed in terms of internal stratigraphical consistency of the OSL ages and/or their geomorphological consistency.

### 5.2. Terrace Ages and River Incision Rates

The formation ages of terrace treads and straths are constrained by dating the overlying loess and fluvial deposits between tread and straths and can be inferred from the OSL ages and/or the deposition rates of the loess and fluvial sediments.

T1: The two fluvial sand samples (L782 and 783) from section B for the T1 terrace were dated to 41.9 ± 2.7 and 39.8 ± 2.5 ka, respectively, and the overlying loess sample (L784) to 29.1 ± 2.1 ka (Figure 2B and Figure 3). These ages are in stratigraphic order. The deposition rate of the silt sediments was calculated to be about 1.1 mm/a for the sediments between the two fluvial samples if errors are not included in the analysis. If this deposition rate is assumed to be constant for the whole overbank silt sediments, the formation age of the terrace tread is calculated to be ~36 ka using the deposition rate of 1.1 mm/a, and the age of the silt at the bottom is induced to be about 43 ka. If the deposition rate is also used for the gravel layer, the age of the terrace strath is inferred to be about 44 ka. The accumulation of the terrace deposits lasts about 8 ka. This is consistent with the period suggested by Weldon [76] and Pazzaglia and Brandon [77]. The tread age of ~36 ka is also constrained by the age (29.1 ± 2.1 ka) of the overlying loess sample (Figure 2B).

T2: The three sand samples (L567, 568 and 569) taken from section C for the T2 terrace (Figure 2C and Figure 3) were OSL dated to 135 ± 10, 137 ± 8 and 134 ± 15 ka, respectively. They are consistent within errors, suggesting that the sand sediments were rapidly deposited. We then deduced that the formation age of the terrace tread is about 135 ka. If we assume that the deposition of the channel gravels was rapid or the deposition and strath carving were simultaneous, the strath age is also inferred to be about 135 ka.

T3: The two samples (L570 and 571) from sand lens within the channel gravel facies in section D for the T3 terrace were from the depths of 9.8 and 4.5 m, respectively, and their OSL ages are respectively 126 ± 9 and 122 ± 6 ka (Figure 2D and Figure 3). The consistency in age between them indicates a higher deposition rate for this terrace. Even so, the deposition rate was calculated to be about 1.3 mm/a based on the ages and the difference in depth between the two samples, and we deduced that the ages of the tread and strath are about 119 and 129 ka, respectively. The deposition of the fluvial sediments occurred during about 10 ka.

T4: The two channel sand samples (L556 and 557) from section E for the T4 terrace (Figure 2F and Figure 3) were determined to be 123 ± 8 and 135 ± 7 ka, respectively. They are not stratigraphically consistent if errors are excluded in the analysis but are in agreement within error limits. We thus deduce that the ages of the tread and strath may be both about 130 ka.

T5: The five samples from section F for the T5 terrace (Figure 2F and Figure 3) were dated, and the two channel sand samples (L572 and 573) were determined to be 139 ± 8 and 139 ± 7 ka, respectively. The overlying overbank samples (L785 and 786) and the top loess sample (L787) were dated to 187 ± 12, 195 ± 12 and 65 ± 4 ka, respectively. The OSL ages of the overbank deposits are larger than those of the underlying channel sands, which can be explained by the age overestimation of the overbank deposits. This is because that the overbank deposits consist largely of reworked bedrock silty-clay pellets which were not well-bleached prior to burial. Therefore, we infer that the strath and the tread ages of this terrace are about 139 ka.

T6: A total of six OSL samples were taken from the two sections (sections G and H) for the T6 terrace (Figure 2G,H and Figure 3). In section G, paleosol immediately overlies the channel gravel layer. The paleosol sample (L558) was dated to 187 ± 8 ka and the overlying loess sample (L559) to 189 ± 12 ka. For section H at about 350 m distance from section G, the two channel sand samples (L561 and 562) were respectively dated to 190 ± 15 and 187 ± 12 ka and the overlying paleosol (L563) and loess (L564) samples to 187 ± 8 and 189 ± 12 ka, respectively. The OSL ages of the six samples are in agreement within errors. The actual difference in ages between them may be masked by their errors. In this case, we deduced that the ages of the tread and strath are about 190 ka.

**Figure 8:**
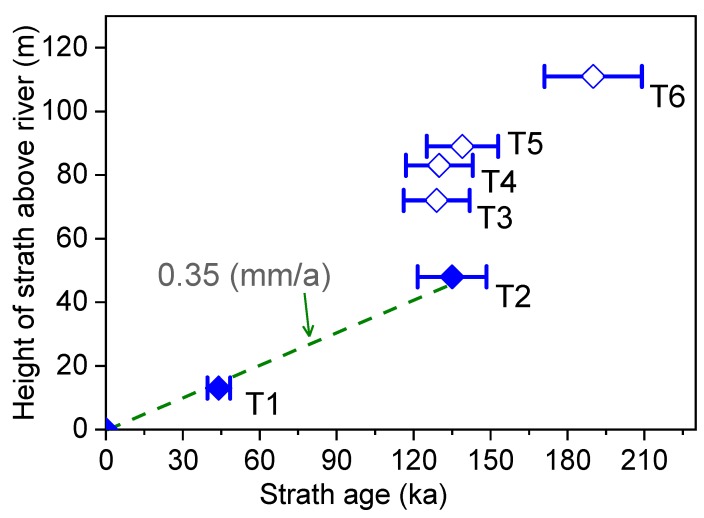
Plot of elevation of strath above the modern river level as a function of strath ages. The error of 10% was assumed for the inferred strath ages. The OSL ages of the T3 to T6 terraces marked as open diamonds are obviously underestimated (see text for details). The dash regression line represents a time-averaged incision rate 0.35 mm/a for the past 135 ka.

As discussed above, the luminescence properties of the samples and stratigraphic consistency of the OSL ages obtained for the terraces appear that the OSL ages are reliable. In order to further evaluate the reliability of the ages, the strath ages obtained for the terraces and strath elevations above the modern river level are plotted in Figure 8. It is known that higher strath terraces are generally formed earlier than lower terraces for a river, implying that the OSL ages of samples from higher terraces should be older than the ages of lower terraces. From Figure 8, it can be seen that the formation ages of the straths for the T2, T3, T4 and T5 terraces are very similar, but their elevations increase from 48 m arl for the T2 terrace to 89 m arl for the T5 terrace. The only reasonable explanation for this situation is that the OSL ages of the fluvial samples from the T3 to T6 terraces were underestimated or regarded as the minimum ages of these terraces.

The elevation of terrace strath above a modern river level is often used to calculate the mean bedrock river incision rates [78,79], which in some cases can be used as a proxy for rock-uplift rates [1]. Although Figure 8 shows that the ages of the T3–T6 terraces were underestimated, the strath heights and ages of the T1 and T2 terraces can be used for calculating the average incision rate for the past 135 ka. The regression of the strath ages versus the elevations for the T1 and T2 terraces above the modern river level defines a mean incision rate of 0.35 mm/a for the past 135 ka (Figure 8). The incision rate for the past 44 ka was calculated to be about 0.35 mm/a by dividing the elevation by the age of the T1 terrace. The incision rate of 0.35 mm/a in this study is similar to the rates of 0.34 mm/a for the past 108 ka in the Heiyukou area [20] and 0.35 mm/ka for the past 70 ka in the Hukou area [19] (Figure 1a). This can be explained by the fact that the Ordos Plateau, an uplifted basin, behaves as a rigid block (Figure 1a). On the other hand, the incision rate also represents the uplift rates of the block.

### 5.3. Implication for Dating Fluvial Terraces

**Figure 9:**
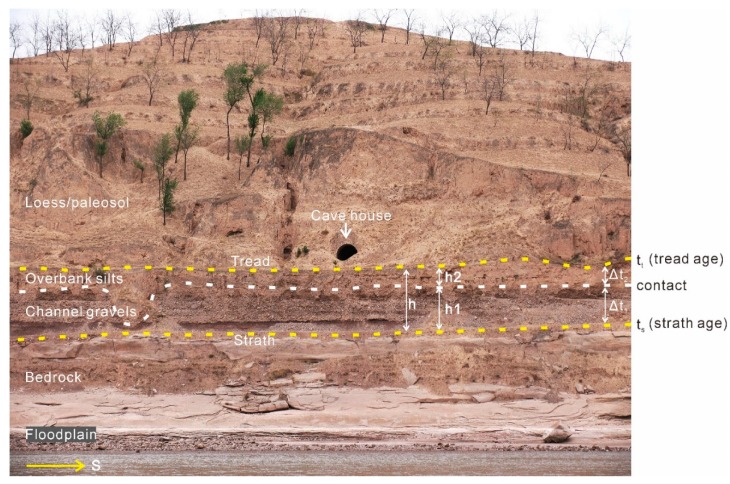
Photograph of an exposure in the Heiyukou area showing the lithostratigraphic units of a fluvial terrace of the Yellow River (flow south (S)) and the boundaries between the units. It also shows the difference (∆) between the formation ages (t_s_ and t_t_) of the strath, and the tread varies from terrace to terrace. h, h1 and h2 represent, respectively, the thickness of the terrace deposits, channel gravels and overbank sediments, h = h1 + h2 (see text for details). The upper overbank facies may be in unconformable contact with underlying channel facies because of erosion or depositional hiatus. The gate (about 2-m-wide) of a cave house excavated in loess provides a scale.

Fluvial terraces are morphostratigraphic units, and terrace deposits are channel sand and/or gravel facies overlain by fine overbank facies deposits bound by bottom strath and upper tread. Loess/paleosol deposits accumulate on the tread in our studied area (Figure 9). Fluvial terraces are geomorphologically classified into strath terraces and fill terraces, and the only difference between them is that there is only a thin layer of fluvial sands or gravels atop bedrock strath surfaces for strath terraces [80]. Practically, different thicknesses (h1 in Figure 9) of the “thin layer” have been used when a terrace is assigned as a strath terrace in the field. The thickness varies from about <3 m [4,77,81,82,83] or <10 m [2,84] or >10 m [85,86,87]. Pazzaglia and Brandon [77] proposed the criterion of the thickness of <3 m of coarse fluvial sediments for strath terraces and considered that this thickness represents the sediments in transport in a channel and the approximate scour depth during bankfull or larger discharges in a river the size of their studied river. This implies that the river downcutting and deposition of channel gravels for a strath terrace are simultaneous, and the thickness of channel gravels for strath terraces is associated with river size.

As shown in Figure 9, both strath age (t_s_) and tread age (t_t_) can be obtained for a terrace. t_s_ refers to the end of an interval of strath formation [88], and t_t_ represents the last time that overbank or channel deposition occurred on the strath. t_s_ is often used to calculate the river incision rate [4,77,78,89] and t_t_ to calculate the age of the deposit and, when displaced by tectonic, the slip rate of faulting [13,90,91] or strath incision rate [90]. As mentioned above, typical terrace deposits include channel gravels unconformably overlying bedrock strath, both generally covered by fine-grained overbank sediments. The overbank deposits may be in unconformable contact with the underlying channel gravels because of erosion; also, when gravels are exposed to air due to river downcutting. The strath ages are often inferred from the ages of the directly overlying fluvial sediments, assuming that the sediments are intimately associated with the beveling of the strath surface and considering that the sediments and the strath have correlative ages [16]. It is noted that the tread ages refer to the timing of abandonment of the tread, not the ages for the deposition of the terrace deposits. The ages can be inferred from the ages of overlying loess and overbank deposits (Figure 9) for some terraces.

Figure 2 shows that the terrace types and the relationship among the ages of strath, tread, fluvial terrace deposits and overlying loess are complicated. This is supported by those for the terrace sections at other localities along the banks of the Jinshan Canyon previously investigated [18,19,20]. Almost all of the terrace deposits for these Yellow River terraces are composed of channel gravels and overbank silts, and these fluvial deposits are mantled by loess/paleosol. Single layers of channel gravels for most of the terraces are >3-m-thick, even up to 20 m, and the thickness of the overbank silts varies from 0 to about 10 m. For the higher terraces (T2–T6 in Figure 3, and the corresponding sections in Figure 2C–F,H) in this study, the OSL ages of the fluvial samples from each section are older than 100 ka and consistent within errors. The consistency may be attributed to the fact that (1) the age differences between samples are less than their errors or (2) the upper age limits of OSL dating for the samples are reached. The above discussion implies that the formation ages of terrace strath and tread can be inferred based on deposition rate and thickness of terrace deposits, regardless of terrace types. Using deposition rates to infer strath ages can overcome the potential age underestimation caused by dating samples from thick-fill terraces [4,77]. For older terraces, the accumulation time of fluvial sediment atop terrace strath may be negligible relative to the formation ages of terrace strath, meaning that the ages of terrace deposits are approximately equal to the formation ages of terrace strath and tread. All the above analyses suggest that terrace deposits should be systemically sampled for dating when dating fluvial terraces. Additionally, the ages of overlying loess deposits are often regarded as strath ages [92,93]. Sections B and F in this study show that the burial ages of loess are much younger than the underlying fluvial deposits, but other sections indicate that the ages of the two types of sediments are almost similar. This implies that whether the ages of terrace loess are approximately equal to the fluvial sediments varies from terrace to terrace. On the other hand, the age of terrace loess can at least be used to constrain the tread age. Here, it should be pointed out that the formation of terrace strath or tread should be specified when we report the formation ages of fluvial terraces.

## 6. Conclusions

In the Baode area in the middle reaches of the Yellow River, seven fluvial terraces (T1 to T7) were identified in the field based on the presence of fluvial sediments and the elevation of terrace straths observed on nine sediment exposures, and the thicknesses of fluvial sediments on these terraces are about 10–16 m. Twenty-five samples for OSL dating using the SAR protocol on quartz grains were collected from the terraces, except for the highest terrace (T7), whose age is beyond the upper limit of OSL dating, and they were dated to ~2–200 ka. The luminescence properties of the samples and stratigraphic consistency demonstrate that the OSL ages obtained appear to be reliable. However, the geomorphologic evidence shows that the ages (>120 ka) of the fluvial samples from the higher terraces (T3 to T6) should be underestimated, because these ages are generally similar to those of the samples from the lower terrace (T2). Based on the deposition rate of fluvial sediments atop the straths, we infer that the formation ages of both terrace strath and tread are, respectively, about 44 ka and 36 ka for the T1 terrace, and the strath and tread of the T2 terrace has the same formation age of about 135 ka. Based on our previous investigations on the Yellow River terrace and the results in this study, we argue that the formation ages of terrace straths and treads calculated using deposition rates of terrace fluvial sediments may be more accurate than those obtained from a single age of one sample. The incision rate was calculated to be about 0.35 mm/ka for the past 135 ka. This is similar to the rates for the past 108 ka in the Heiyukou area and for the past 70 ka in the Hukou area in the middle reaches of the river, implying the uplift rates of the Ordos Plateau as a rigid block.

## Figures and Tables

**Table 1:** The results of optical dating of the samples from the Yellow River terraces in the Baode area.

Section No.	Lab Code	Field No.	Depth (m)	Sediment	Grain Size (µm)	K * (%)	K ** (%)	U ** (ppm)	Th ** (ppm)	Water Content ^#^ (%)	Dose rate (Gy/ka)	No. of Aliquots Measured	Arithmetic Mean De (Gy)	Central Age Modeling (CAM)		
														OD^+^ (%)	Mean De (Gy)	Age (ka)
**Terrace T0**																
A	L552	BD-OSL06	0.98	Overbank silt	4–11	1.8	1.87 ± 0.10	2.36 ± 0.10	9.81±0.24	10	3.30 ± 0.12	3	30.4 ± 1.1		30.5 ± 1.2	9.2 ± 0.5
	L551	BD-OSL05	2.05	Channel sand	90–125	1.72	1.63 ± 0.09	2.02 ± 0.09	10.10 ± 0.22	5	2.89 ± 0.08	21	6.7 ± 1.5	72	5.0 ± 0.8	1.7 ± 0.3
	L550	BD-OSL04	3.30	Channel sand	150–250	2.98	3.25 ± 0.11	0.68 ± 0.06	5.52 ± 0.17	5	3.69 ± 0.10	25	13.5 ± 2.4	69	10.0 ± 1.5	2.7 ± 0.4
	L549	BD-OSL03	4.77	Channel sand	150–250	2.35	2.32 ± 0.10	0.83 ± 0.07	5.63 ± 0.16	5	2.84 ± 0.09	21	53.7 ± 5.5	53	47.4 ± 5.6	16.7 ± 2.0
**Terrace T1**																
B	L784	BD06-OSL03	2.40	Loess	4–11	1.4		3.01 ± 0.37	11.41 ± 1.24	10	3.12 ± 0.15	4	91.3 ± 5.4		90.7 ± 4.8	29.1 ± 2.1
	L783	BD06-OSL02	8.80	Channel sand	150–250	1.85		2.48 ± 0.33	9.13 ± 1.10 *	5	2.95 ± 0.11	21	122.7 ± 7.2	22	117.6 ± 6.0	39.8 ± 2.5
	L782	BD06-OSL01	11.20	Channel sand	150–250	1.65		2.01 ± 0.37	12.24 ± 1.24 *	5	2.85 ± 0.11	21	125.2 ± 7.2	21	119.5 ± 6.3	41.9 ± 2.7
**Terrace T2**																
C	L567	BD-OSL21	1.05	Overbank silt	90–125	1.72	1.68 ± 0.10	1.59 ± 0.07	8.75 ± 0.22	10	2.64 ± 0.09	16(2)	378.9 ± 65.8	21	357 ± 24	135 ± 10
	L568	BD-OSL22	2.00	Overbank silt	90–125	1.72	1.64 ± 0.10	1.90 ± 0.09	8.02 ± 0.20	10	2.60 ± 0.09	13	369.4 ± 17.6	12	355 ± 18	137 ± 8
	L569	BD-OSL23	2.70	Overbank silt	90–125	1.72	1.72 ± 0.10	1.80 ± 0.08	7.32 ± 0.20	10	2.59 ± 0.09	8	366.0 ± 41.8	29	346 ± 38	134 ± 15
**Terrace T3**																
D	L571	BD-OSL25	4.8	Channel sand	150–250	2.2	2.35 ± 0.09	1.09 ± 0.08	6.49 ± 0.18	5	2.98 ± 0.08	20	366.9 ± 14.5	13	365 ± 13	122 ± 6
	L570	BD-OSL24	9.8	Channel sand	90–150	1.88	1.09	1.70 ± 0.10	8.62 ± 0.21	5	2.73 ± 0.09	22	361.8 ± 25.5	29	344 ± 23	126 ± 9
**Terrace T4**																
E	L557	BD-OSL11	4.80	Channel sand	125–150	1.8	1.80 ± 0.09	1.23 ± 0.07	5.83 ± 0.17	5	2.50 ± 0.08	23	357.3 ± 21.0	23	339 ± 15	135 ± 7
	L556	BD-OSL10	16.2	Channel sand	150–250	2.2	2.10 ± 0.09	0.80 ± 0.07	5.03 ± 0.16	5	2.51 ± 0.08	23	321.1 ± 20.3	26	310 ± 18	123 ± 8
**Terrace T5**																
F	L787	BD06-OSL06	12.9	Loess	4–11	1.95		2.62 ± 0.40	13.19 ± 1.33 *	10	3.55 ± 0.17	4	228.3 ± 3.2		228.9 ± 6.6	65 ± 4
	L786	BD06-OSL05	16.00	Overbank silty clay	4–11	1.65		2.01 ± 0.35	10.89 ± 1.18 *	10	2.93 ± 0.14	3	578.0 ± 20.4		570 ± 23	195 ± 12
	L785	BD06-OSL04	22.20	Overbank silty clay	4–11	1.65		2.37 ± 0.35	10.72 ± 1.18 *	10	2.99 ± 0.14	4	590.1 ± 53.6		559 ± 26	187 ± 12
	L573	BD-OSL27	23.00	Channel sand	90–125	2.67	2.69 ± 0.10	1.00 ± 0.08	6.42 ± 0.18	5	3.27 ± 0.09	52	481.9 ± 20.6	24	456 ± 19	139 ± 7
	L572	BD-OSL26	25.7.00	Channel sand	90–125	2.35	2.45 ± 0.09	1.18 ± 0.08	11.50 ± 0.25	5	3.43 ± 0.08	25	509.3 ± 35.3	23	476 ± 26	139 ± 8
**Terrace T6**																
G	L559	BD-OSL13	3.10	Loess	4–11	1.72	1.78 ± 0.09	2.57 ± 0.09	9.67 ± 0.22	10	3.21 ± 0.12	5	612.8 ± 25.0		607 ± 32	189 ± 12
	L558	BD-OSL12	7.00	Paleosol	4–11	1.96	2.07 ± 0.09	2.07 ± 0.09	11.50 ± 0.25	15	3.26 ± 0.12	5	642.9 ± 38.0		609 ± 17	187 ± 8
H	L564	BD-OSL18	1.00	Loess	4–11	1.88	1.73 ± 0.10	1.88 ± 0.09	9.15 ± 0.22	10	3.00 ± 0.12	6	593.1 ± 37.1		571 ± 29	191 ± 12
	L563	BD-OSL17	5.15	Paleosol	4–11	2.04	2.23 ± 0.10	2.00 ± 0.08	9.60 ± 0.22	15	3.27 ± 0.12	6	605.1 ± 20.3		623 ± 24	191 ± 10
	L562	BD-OSL16	7.10	Channel sand	150–250	2.2	2.32 ± 0.09	0.96 ± 0.07	5.64 ± 0.16	5	2.84 ± 0.08	21	547.0 ± 28.4	23	531 ± 29	187 ± 12
	L561	BD-OSL15	9.10	Channel sand	150–250	2.2	2.10 ± 0.09	0.80 ± 0.07	4.39 ± 0.15	5	2.50 ± 0.08	25	497.6 ± 32.2	31	474 ± 31	190 ± 15

*: Determined using flame photometry; **: Determined using neutron-activation-analysis (NAA), except for samples L782–787, for these six samples whose U and Th contents were determined using the alpha counting method; the K contents obtained using NAA were used for dose rate calculation, except for samples L782–787 (for these six samples, the K contents obtained using flame photometry were used, and a relative uncertainty of 5% was assumed); OSL: optically stimulated luminescence; ^#^: Relative uncertainty of 20% is assumed for water contents for all samples; ^+^: OD = overdispersion; for fine grains, the OD value is not calculated. All uncertainties are reported as one sigma.
